# Potential bioactivity of Phoenix dactylifera fruits, leaves, and seeds against prostate and pancreatic cancer cells

**DOI:** 10.3389/fnut.2022.998929

**Published:** 2022-10-28

**Authors:** Hesham S. Ghazzawy, Mostafa M. Gouda, Nabil S. Awad, Nadi Awad Al-Harbi, Mesfer M. Alqahtani, Maha M. Abdel-Salam, Mohamed A. Abdein, Sanad M. Al-Sobeai, Asmaa A. Hamad, Hassan M. Alsberi, Gamal A. Gabr, Dalia M. Hikal

**Affiliations:** ^1^Date Palm Research Center of Excellence, King Faisal University, Al Ahsa, Saudi Arabia; ^2^Central Laboratory for Date Palm Research and Development, Agriculture Research Center, Giza, Egypt; ^3^College of Biosystems Engineering and Food Science, Zhejiang University, Hangzhou, Zhejiang, China; ^4^Department of Nutrition and Food Science, National Research Centre, Giza, Egypt; ^5^Department of Genetics, Faculty of Agriculture and Natural Resources, Aswan University, Aswan, Egypt; ^6^College of Biotechnology, Misr University for Science and Technology, Giza, Egypt; ^7^Biology Department, University College of Tayma, University of Tabuk, Saudi Arabia; ^8^Department of Biological Sciences, Faculty of Science and Humanities, Shaqra University, Ad-Dawadimi, Saudi Arabia; ^9^Department of Pomology, Faculty of Agriculture, Assiut University, Assiut, Egypt; ^10^Department of Biology, Faculty of Arts and Science, Northern Border University, Rafha, Saudi Arabia; ^11^Sajir College of Arts and Science, Shaqra University, Sharqa, Saudi Arabia; ^12^Department of Biology, Faculty of Science, Taif University, Taif, Saudi Arabia; ^13^Department of Botany and Microbiology, Faculty of Science, Cairo University, Giza, Egypt; ^14^Department of Basic Medical Science and Histopathology, National Organization for Drug Control and Research, Giza, Egypt; ^15^Department of Pharmacology and Toxicology, College of Pharmacy, Prince Sattam Bin Abdulaziz University, Al-Kharj, Saudi Arabia; ^16^Agricultural Genetic Engineering Research Institute (AGERI), Agricultural Research Center, Giza, Egypt; ^17^Nutrition and Food Science, Department of Home Economics, Faculty of Specific Education, Sohag University, Sohag, Egypt

**Keywords:** functional food, cytotoxic effect, apoptosis, pancreatic prostate, cancer cells line, date palm

## Abstract

The use of functional foods’ phytochemicals in the chemoprevention of different cancer diseases has become one of the hot scientific areas in the clinical nutrition field. For instance, the Khalas palm cultivar (KPC; *Phoenix dactylifera*) is one of the natural sustainable resources that have high bioactivity and functionality. This study aimed to investigate the antiproliferative activity and mode of action of KPC’s different parts on prostate (Pc3) and pancreatic (panc1) cancer cells at a molecular level. In the methods, KPC’s leaves, seeds, and fruits’ chemical composition and phytochemical analysis were analyzed. Also, the cytotoxic effects of each extract were assessed against pc3 and panc1 cell lines. Besides, induction of apoptosis, cell cycle analysis, and gene expression of both Cap3 and Cap9 were studied. The obtained results indicated that KPC leaves extract exhibited the highest significant (*P* < 0.01) anti-proliferation activity against the utilized cancer cell lines compared to fruits and seeds extracts. Also, there were significant (*P* < 0.05) differences in the phenolic contents, flavonoid of compounds, and antioxidant power of the leaves when compared to the seeds and fruits. Additionally, the highest cytotoxic effect (lowest IC_50_) was recorded with leave extract than seeds and fruits. Meanwhile, the seeds extract induced (*P* < 0.05) the apoptosis and arrested cells in the G2/M phase as well as up-regulated the gene expression of the apoptotic-related genes (Casp3 and Casp9) compared to the control group. In conclusion, this study showed that the presence of bioactive components in the KPC different parts extracts have the significant ability to induce the apoptotic pathway that could down-regulate the proliferation of prostate (pc3) and pancreatic (panc1) cancer cells. The pathway mechanism of action was induced by the phytol molecule presented in its leaves extract.

## Introduction

Cancer is one of the significant causes of global mortality and morbidity and remains a compelling challenge for public health (1, 2). For instance, prostate cancer is the most common cancer in men after lung cancer (3). Rawla (4) reported that 1,276,106 new patients were recorded with prostate cancer with a 3.8% death count in men during the year 2018. Prostate cancer rates are increasing rapidly in most countries (5). Meanwhile, pancreatic cancer is considered an uncontrollable malignant tumor. Such cancer cells are the major cause of global cancer fatalities in developed and developing countries alike, including the United States, Egypt, and Saudi Arabia (4).

There are several treatment kinds for cancer diseases (2, 6). These treatments include surgery, chemotherapy, radiotherapy, and immunotherapy. Such treatments have many different failure conditions due to adverse reactions, drug resistance, side effects, and drug specificity (7, 8). Also, it is noteworthy that cancer prevention and therapy had been improved significantly by using phytochemical compounds, such as phenolics and flavonoid compounds (9–11). These organic compounds that represent above 50% of all modern clinical drugs have been discovered from plants and microorganisms (12). Moreover, functional foods including some of the commonly consumed herbs [plant leaves (13)] phytochemicals have significant antioxidant capacities that could act against cancer cell proliferation and differentiation (14–16). Rodríguez et al. (17) stated that natural origin therapeutics, which are rich in phenolics and flavonoid contents considered to be potent preventive drugs against cancer cells by their anti-apoptotic regulating functional groups.

The date palm tree (*Phoenix dactylifera* L.) belongs to the Arecaceae family and is an important crop in many regions of the world due to its overall nutritional and pharmacological effects, which have valuable components (18). There are several palm cultivators in the Middle East and North African regions such as Khalas (KPC), Riziz, Shish, Barhee, Ajwa, Anbra, and Mejdol palm date. From these varieties, the total number of KPC is about (760,476,7) trees (19, 20). Additionally, Khalas date is mainly grown and manufactured in significant amounts in Saudi Arabia’s eastern provinces, such as Al-Hassa, Al Kharj, and Qaseem (21). It has several secondary metabolites that act as high antioxidant, anticancer (22), hepatoprotective (23), neuroprotective, nephroprotective (24), gastrointestinal protective (25), antidiabetic (26), antihyperlipidemic (27), sexual improvement (28), and antimicrobial potentials (29, 30). Moreover, *In vitro* trials were done to evaluate the anticancer activity of KPC extracts toward different cancer cell lines such as human epithelial colorectal adenocarcinoma (Caco-2) (31), the human melanoma-derived cell line (IGR-39) (32), ehrlich ascites carcinoma (33), and hepatocellular carcinoma (34). For instance, Rahmani et al. (35) found that KPC fruit extract reduced prostate cancer development. Additionally, the fruits contain the least amount of moisture and maintain a delicate texture and sweet taste in the last stage of ripe dates, characterized by good storage capacity. Mineral content in dates has been measured in a variety of studies. According to a recent study, the concentration ranges for Mn, Zn, Cu, and Se (percent mg) in dates from Bangladesh were 0.85–1.1, 0.69–0.72, 0.32–0.36, and 0.22–0.31, respectively (36). In prior research of five date cultivars (Ajwa, Beid, Burni, Rabia, and Safawi), potassium was shown to be the most prevalent macroelement (0.185–1.51%) in all of the dates studied. The concentrations of Zn and Cu were found to be between 27.5 and 72.70 ppm and 4.95 and 6.25 ppm, respectively (37). Thus, it can work as an effective anticancer agent. However, the actual preventive mechanism of its components is still not evident. Therefore, several reports have recommended further in-depth studies to understand the KPC biological, therapeutic, and nutritive roles of at the cellular and molecular levels (15, 22, 38).

The present study aimed to evaluate the antiproliferative impact of KPC’s leaves, seeds, and fruit extracts against pancreatic and prostate cancer cell lines. Thus, to provide evidence of the applicability of KPC extracts as bioactive anticancer agents based on their unique chemical structures for its potential application in the clinical nutrition field.

## Materials and methods

### Chemicals, materials, and cells

Ethanol of HPLC grade was purchased from Sigma-Aldrich^®^ solutions (Merck, USA). Folin-Ciocalteu reagent was purchased from Aladdin Reagent Co., Ltd., China. Deionized water was used. Fetal bovine serum (FBS), Dulbecc’s modified Eagle medium (DMEM), phosphate-buffered solution (PBS), penicillin/streptomycin (P/S), and 3-(4,5-dimethylthiazol-2-yl)-2,5-diphenyltetrazolium bromide (MTT) were purchased from Life Technologies (Carlsbad, CA, USA).

Leaves, fruits, and seeds of the Khalas palm cultivar (KPC, *Phoenix dactylifera* L.) were collected from Alahsaa, Saudi Arabia (GPS coordinates: Latitude: 25°25′27.59′′N, Longitude: 49°37′11.39′′E). Collected plant materials were washed in distilled water and dried at room temperature for several days.

Cells of pancreatic cancer (Panc1) and prostate cancer (Pc3) were purchased from the Holding Company for Biological Products and Vaccines, Egypt VACSERA).

### Samples collection and extract preparation

The air-dried plant materials were cut into small pieces and dried in shade for 2 days until they were completely dried, then pulverized into fine powder. Next, 500 g of powdered materials were packed in the soxhlet apparatus extraction unit (Soxtherm, Gerhardt, Germany) and successive extraction was performed using ethanol solvent. The solution of the extract was filtered through Whatman filter paper no. 1 and concentrated using a rotary flash evaporator and dried under a vacuum condition.

### The chemical composition measurements

The general chemical composition [moisture (%), fiber (%), fats (%), protein (%), total reducing sugars (%), fructose (%), glucose (%), sucrose (%) of the samples were analyzed using standard methods of the Association of Official Analytical Chemists] (39) at Date Palm Research and Development, Agriculture Research Center, Giza, Egypt. Also, Mn, Cu, and Zn were analyzed by using inductively coupled plasma (ICP) (iCAP 6000 Series, Thermo Scientific, MA, USA).

### Determination of total phenolic contents

The total phenolic contents were determined by the Folin–Ciocalteu method as described by Pieme et al. (40) with some modification at Food Technology Research Institute, Agriculture Research Centre, Giza, Egypt. In the mixture, 180 μL diluted extract, 750 μL diluted Folin Ciocalteu reagent, and 7.5% of 2 mL sodium carbonate were added together to make a substance. After mixing the substance, 7 mL of deionized water was added. After that, put the mixture in the dark at ambient conditions for 2 h so that the substance can finish its reaction. It should be noted that 765 nm absorbance was recorded. Moreover, Garlic acid was referred to as standard. The units were mg gallic acid (GAE; 5–100 μg/mL).

### Determination of total flavonoid contents

The total flavonoids were measured using the aluminum chloride colorimetric method according to Charoensin (41) at Food Technology Research Institute, Agriculture Research Centre, Giza, Egypt. One hundred μl were added together with 1.5 ml of 95% ethanol, 100 μL of 10% AlCl_3_, 100 μl of 1 M potassium acetate, and 2.8 ml of deionized water in each extract. It should be noted that the absorbance of the substance is found to be 415 nm. The unit of total flavonoid content was milligram quercetin equivalent per gram extract (mg QE/g extract).

### Determination of antioxidant activity

The antioxidant activity of each extract was detected according to Bray et al. (42). Methanolic extracts in various concentrations (100–500 μL) were mixed separately with 2.5 mL of 0.2 mM PBS (pH 7.4) and 2.5 mL of potassium ferricyanide (1% w/v). Each mixture was incubated at 50°C for 20 min. Then, 2.5 mL of trichloroacetic acid (10% w/v) was added and centrifuged at 3,500 rpm for 8 min, followed by 2.5 mL of distilled water and later 0.5 mL of ferrous chloride (0.1% w/v). The absorbance at 700 nm was estimated. As a positive reference standard, ascorbic acid was utilized.

### Separation of the phytochemicals by GC/MS

The crude extracts were analyzed using Shimadzu GCMS-QP2010 Plus, Tokyo, Japan following the method of El Sherif et al. (43) with some modifications at Department of Nutrition and Food Science, National Research Centre, Dokki, Giza, Egypt. In brief, the mobile phase (helium; 99.99%) was used at a flow rate of 1 mL/min. The instrument was equipped with a capillary column DB-5MS (30 m length, 0.25 mm thickness, 0.25 m diameter). A 1 μL sample was injected into the split/splitless inlet in splitless mode at 260°C. The temperature programming of the GC/MS method analysis starts with the oven temperature. 60°C for 5 min, then increased by 10°C/min to 240°C for 15 min. the temperature of the interface was 220°C and the ion source 200°C. The range of scan mode (50–550 amu) was used for data acquisition. The relative amount of each constituent was calculated by measuring the corresponding peak area and represented as a percentage of the sum of areas of all peaks.

### Cancer cell lines and culture

Cells of pancreatic cancer (Panc1) and prostate cancer (Pc3) were suspended in a medium containing 10% fetal bovine serum (RPMI 1640) and incubated at 37°C in atmospheric 5% CO_2_.

### Detection of cell viability

The MTT cell viability assay was utilized to explore the activity of each extract against pancreatic (Panc1) and prostate (Pc3) cancer cell lines. MTT assay relies on the dehydrogenase enzyme of a living cell to split the MTT tetrazolium yellow rings to make an unsolvable dark blue formazan crystal. This crystal was impermeable to cell membranes that cause its growth in healthy cells. The solubility of formazan’s dark blue color depends on the count of living cells. It was found that dwindling in MMT measured to be 570 nm approximately. The used cell lines were plated in serum-free culture media in a flat bottom 96 well microplate 0.5 × 10^5^ cell/well and used with multiple concentrations ranging from 312.5 to 10,000 μg/mL of each extract in a mummified 5% CO_2_ atmosphere at 37°C for 48 h. After the incubation, the media was removed. Each time 40 μL of MTT solution/well was included. The plates were swung and then the solution kept at room temperature for 4 h. A microplate ELISA reader was used to calculate the absorbance at 570 nm. Three replicates were measured for each sample. Data were presented as a relative viability% as follows:


Cellviability(%)=[(ODoftreatedcells/(ODofcontrol]×100

Growthinhibition(%)=100-Cellviability(%).

Then, the IC_50_% was calculated from the dose-dependent curve.

### Induction of apoptosis and cell cycle analysis

Apoptosis and necrosis induction was investigated by Annexin V-FITC/PI apoptosis assay according to Pumiputavon et al. (44). Cell lines at 1 × 10^5^ cells/mL were fixed in 24-well plates under IC_50_% methanolic concentration for 24 h in each extract. The population of apoptotic cells was calculated by Annexin V-FITC (fluorescein isothiocyanate)/P.I. (propidium iodide) co-staining assay. After 24 h of incubation, the cells were collected and then passed through the centrifugal process at 1,800 rpm for 8 min. The pellet was resuspended in a 50 μL binding buffer containing 0.5 μL Annexin V-FITC and then incubated at 4°C for 30 min in the dark. Propidium iodide (PI) (Sigma-Aldrich, USA) (50 μg/mL) in 200 μL binding buffer was supplemented in each tube and then incubated for 5 min. Lastly, flow cytometry was used to further investigate the cell.

For cell cycle analysis, cells (1 × 10^5^ cells/mL) were treated with the IC_50_% concentration of each extract for 24 h as previously described by Azadmehr et al. (45). Cells were centrifuged and fixed in 70% ethanol. After washing, cells were resuspended in 1 mL PBS containing 10 mg/mL RNase and 1 mg/mL PI (Sigma-Aldrich, USA). After that, these cells were incubated for 1 h at 37°C in the dark. Lastly, the cells were examined on a FACScalibur flow cytometer (BD Bioscience Ltd. Co., Canada).

### Expression analysis of apoptotic-related genes

The expression of apoptosis-related genes, caspase-3 (CASP3) and caspase-9 (CASP9) were analyzed using real-time PCR according to Fard et al. (22). The cell lines were treated with each extract and controlled at established IC_50_ concentration. Thereafter, RNA was isolated using the Trizol reagent according to manufacturer instructions. The concentration and purity of the RNA samples were verified on an agarose gel and by the A260/A280 ratio using a NanoDrop Spectrophotometer. RT-qPCR was performed by the use of SYBR^®^ Green dye (One-Step RT-PCR Kit, Bio-Rad) on a Rotor-Gene Q real-time PCR cycler. The sequences of used primers for amplification are as follows:

Caspase3-F 5′-TTC ATT ATT CAG GCC TGC CGA GG-3′ and Caspase3-R 5′-TTC TGA CAG GCC ATG TCA TCC TCA-3′, Caspase 9 F 5′-CGAACTAACAGGCAAGCAGC-3′, and Caspase 9 R 5′-ACCTCACCAAATCCTCCAGAAC-3′ and GAPDH F 5′-GTCTCCTCTGACTTCAA-3′ and GAPDH R 5′-ACCACCCTGTTGCTGTA-3′.

The PCR cycling conditions for caspases-9 and -3 were as follows: amplification at 95°C for 1 min, denaturation for 30 cycles at 95°C for 15 s, annealing at 56.6°C, and 57°C for GAPDH for 15 s, primer extension at 72°C for 10 s, and final extension at 72°C for 7 min. Additionally, the quantitative analysis measured the threshold cycle (CT) values in exponential phase amplification, and ΔCT was calculated from the difference between CT values of Casp3, Caspase9, and GAPDH gene. Then, 2^–ΔΔCt^ was calculated from the fold changes of treated cells in correlation with untreated cells.


DDCT=DCTE-DCTC

Where, ΔCTE is the difference between the CT values of the Casp3, Cas9, and the CT is the value of the GAPDH gene of the treated cells.

### Statistical analysis

Experiments were conducted in three replicates and the mean of values ± standard deviation (SD) were used in the figures and table. One-way analysis of variance (ANOVA) by using SPSS 20.0 (Chicago, IL, USA) was used for the statistical measurements, and *P* < 0.05 (*) was considered statistically significant and *P* < 0.01 (^**^) was considered highly significant. In which, Least significant differences (LSD) have been calculated to measure the signification among the tested groups. Also, IC_50_ was calculated based on the regression equation.

## Results

### *Phoenix dactylifera* (KPC) chemical composition

**FIGURE 1:**
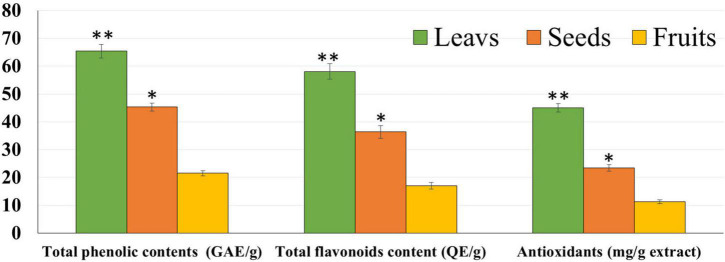
Total phenolic contents, flavonoid, and antioxidants of leaf, seed, and fruit ethanolic extracts. The highest antioxidant potential was observed with leaf extract followed by seed and fruit extracts. *P-value was considered statistically significant (*P* < 0.05) and **P-value was considered highly significant (*P* < 0.01).

The chemical composition of the different parts of the date cultivar (KPC) was investigated to better explain the observed biological activity of these different parts of the date palm on human cancer cell lines. In Table 1, it has been observed that the KPC date cultivar was very low in sucrose content with a high percentage of fiber (2.33%) and protein contents (1.175%). The total contents of phenols and flavonoids of leaves, seeds, and fruits’ ethanolic extracts are evaluated and illustrated in Figure 1. The highest concentration of total phenolic contents and flavonoids was observed in leaves extract followed by seeds extract, while the lowest concentration was noted in the fruit extract. Several previous studies showed the high carbohydrate content of dates (approximately 70–80%), making them a quick and bountiful energy source. Moreover, date fruits are low in starch and high in protein content ranging between (1.03 and 5.60%), dietary fiber (6.40–11.50%), and the amount of minerals (0.10–916 mg/100 g dry weight), and also contains a group of specific vitamins such as (C, B1, B2, B3, and A) (46, 47).

**TABLE 1:** Chemical characterization of the used Khalas date.

Elements analysis	Khalas date (KPC)
Moisture (%)	15.88 ± 0.90
Fiber (%)	2.33 ± 0.20
Fats (%)	0.11 ± 0.01
Protein (%)	1.175 ± 0.02
Nitrogen (%)	0.185 ± 0.01
Total reducing sugars (%)	66.75 ± 2.04
Fructose (%)	30.98 ± 2.56
Glucose (%)	35.77 ± 1.90
Sucrose (%)	BLD
Mn	2.88 ± 0.001
Cu	1.15 ± 0.025
Zn	1.41 ± 0.004

BLD, below detection limit. The values are the mean ± standard deviation of three replicates.

**FIGURE 2:**
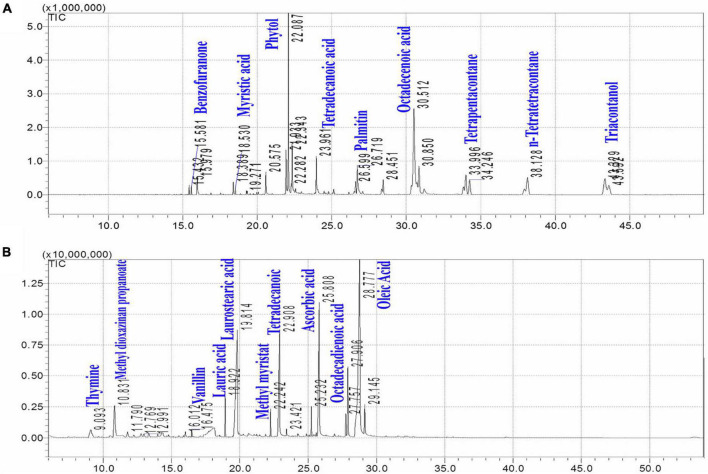
Chemical separation of Khalas leaves **(A)** and seeds **(B)** by GC/MS/MS.

Dates vary in chemical composition based on many factors, including the type of variety and soil conditions of different moisture contents and salts. There is also a role for agricultural procedures and the stage of maturity (48–50). Many variations were identified, such as color, texture, and taste/flavor. The texture of unripe green dates (chimera stage) is firm, with the highest moisture content and tannin acid. Dates begin to lose moisture and produce large amounts of sugar when they reach complete, colorful ripeness (Besser) (51). In addition, moisture loss accelerates in the soft brown (wet) phase, the fruits become smoother in texture, and sucrose turns into inverted sugars. The wet stage of dates is the most attractive because it is the softest and sweetest. Meanwhile, the separation of the extracts by GC/MS showed that the leaves had more compounds than seeds extract with a very high percentage of phytol and octadecenoic acid (Figure 2). A higher percentage of the phytochemicals was achieved in the leaf parts of the KPC tree. Also, the high presence of phytol in the leaves was observed compared to the seed extract.

### Cell viability

**FIGURE 3:**
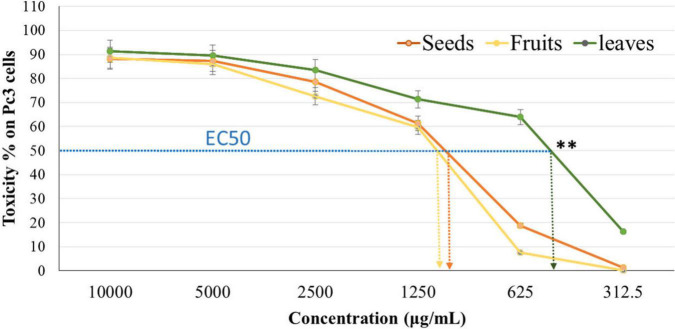
Cytotoxic effect of leaf, seed, and fruit extract on prostate cancer cells (Pc3). ^**^P-value was considered highly significant (*P* < 0.01).

**FIGURE 4:**
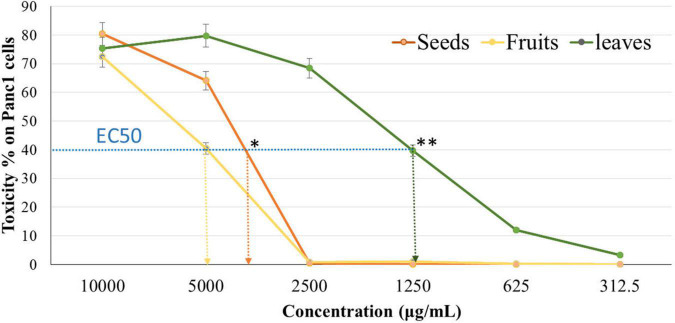
Cytotoxic effect of leaf, seed, and fruit extract on pancreatic cancer cells (Panc1). *P-value was considered statistically significant (*P* < 0.05) and **P-value was considered highly significant (*P* < 0.01).

The cytotoxicity of Panc1 and Pc3 cells was significantly increased following incubation with the ethanolic extracts of leaves, seeds, and fruit parts. Inhibition of Panc1 and Pc3 cell proliferation by these extracts was dose-dependent (Figures 3, 4). The Pc3 IC_50_ of leaves extract was the lowest (*P* < 0.01) with 475 μg/mL compared to both seeds with 950 μg/mL and fruits with 985 μg/mL. Meanwhile, the seeds Pnc1 IC_50_ was significantly (*P* < 0.05) lower than fruits extract, respectively.

### Apoptosis and cell cycle analysis

**FIGURE 5:**
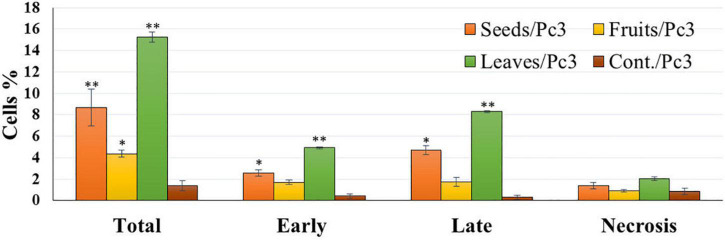
Percentages of apoptotic and necrotic cells in and Pc3 cell population after treatment with leaf, seed, and fruit extract separately. *P-value was considered statistically significant (*P* < 0.05) and **P-value was considered highly significant (*P* < 0.01).

**FIGURE 6:**
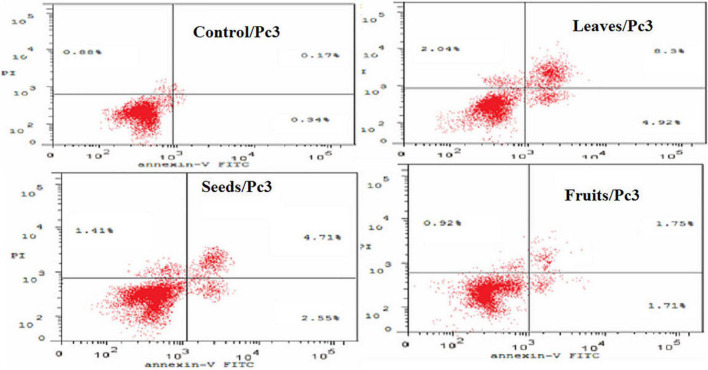
Induction of apoptosis after treatment of Pc3 cells with leaf, seed, and fruit extracts separately in comparison with control.

**FIGURE 7:**
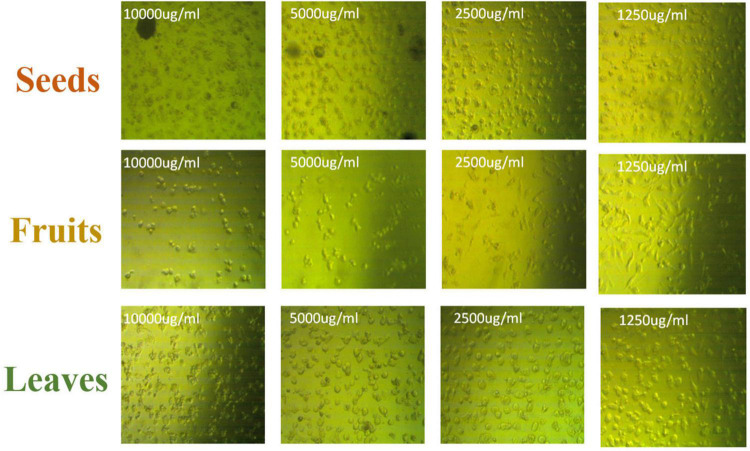
Pc3 cytotoxic morphology after the treatment with leaf, seed, and fruit extracts separately in comparison with control.

**FIGURE 8:**
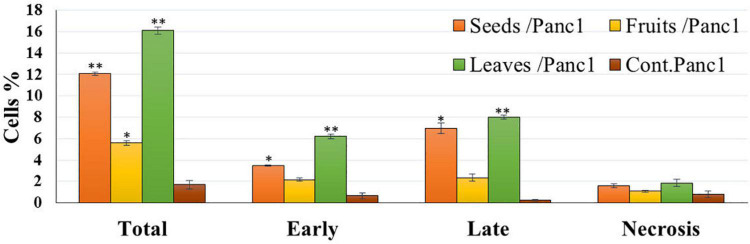
Percentages of apoptotic and necrotic cells in and Panc1 cell population after treatment with leaf, seed, and fruits extract separately. *P-value was considered statistically significant (*P* < 0.05) and **P-value was considered highly significant (*P* < 0.01).

**FIGURE 9:**
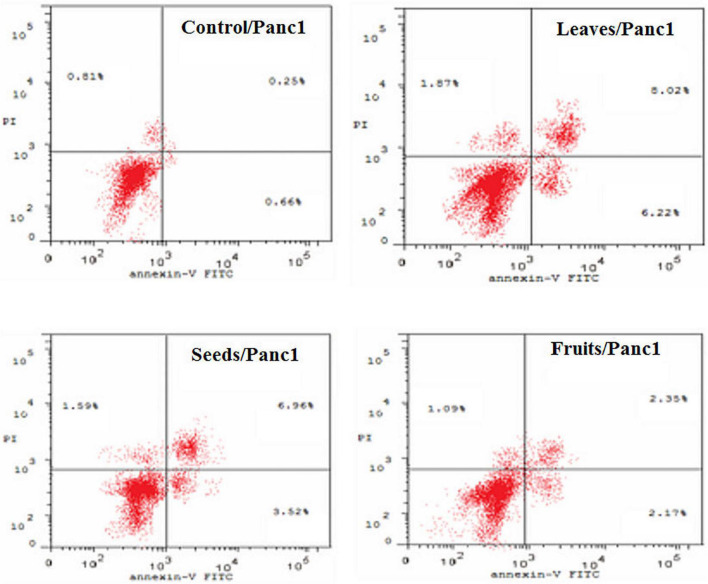
Induction of apoptosis after treatment of panc1 cells with leaf, seed, and fruit extracts separately in comparison with control.

**FIGURE 10:**
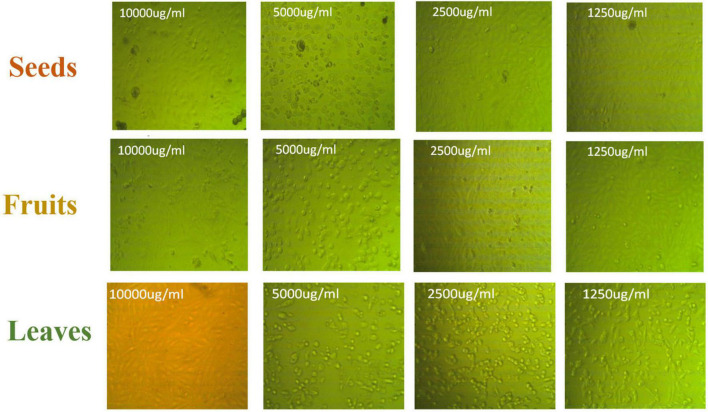
Panc1 cytotoxic morphology after the treatment with leaf, seed, and fruit extracts separately in comparison with control.

The apoptotic effect of the examined extracts was investigated by Annexin-FITC/propidium iodide double staining flow cytometric assay. Obtained results showed that all used cancer cell populations tend to shift from viable to apoptotic in comparison with the control group. Moreover, the late apoptosis events were increased than early apoptotic events in all treatments with all cancer cell lines, as shown in Figures 5–10. The apoptotic populations increased significantly in the Pc3 cell line due to the treatment with the extracts when compared with the control cells. But the apoptosis percent was increased significantly than necrosis in all treatments of utilized cancer cells, as shown in Figures 5, 6. Also, the morphological images of Pc3 have shown that all 1,250–10,000 μg/mL concentrations of the leaves extract had very high cytotoxic effects compared to the 1,250 and 2,500 μg/mL fruit extract with lower cytotoxic effects (Figure 7).

Figures 8, 9 show the apoptotic effect of the different extracts on Panc1 compared to the control group. It has been observed that the leaves extract was significantly highest (*P* < 0.01) compared to the control group. On the other hand, the necrosis had no significant changes among the different groups. Additionally, the concentration of 5,000 μg/mL from seed extract showed high cytotoxic effects on the Panc1 cell lines (Figure 10).

**FIGURE 11:**
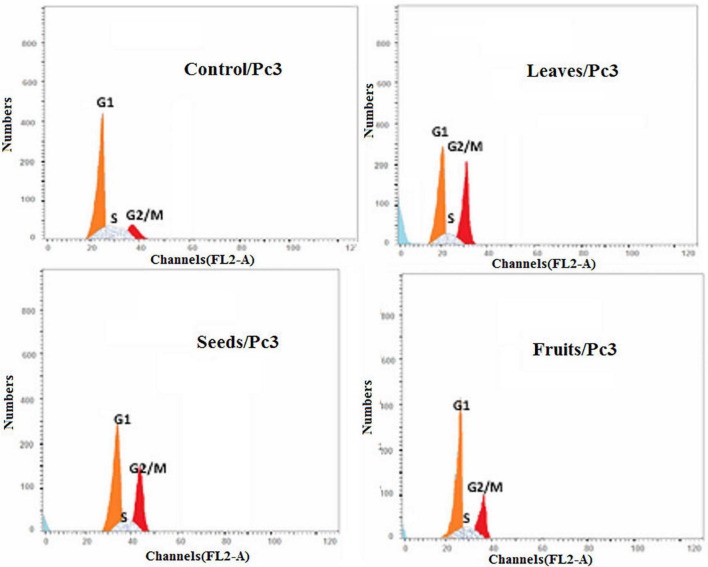
Cell cycle analysis after treatment of Pc3 cells with leaf, seed, and fruit extracts separately in comparison with control.

**FIGURE 12:**
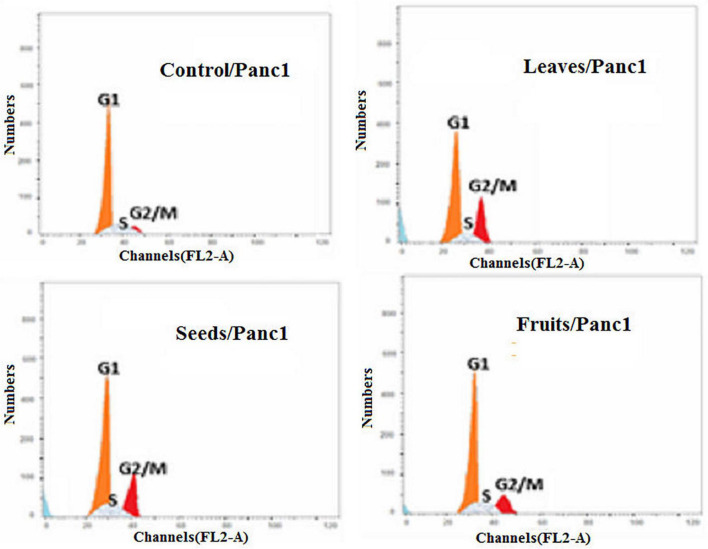
Cell cycle analysis after treatment of Panc1 cells with leaf, seed, and fruit extracts separately in comparison with control.

Moreover, the effect of each extract on the cell cycle progression of both Pc3 and Panc1 cancer cell lines showed that the treatments caused cell growth arrest at the G2/M phase (Figures 11, 12). The highest effects were presented by leaf extracts, followed by seed and fruit extracts.

### Expression analysis of related apoptotic genes

**FIGURE 13:**
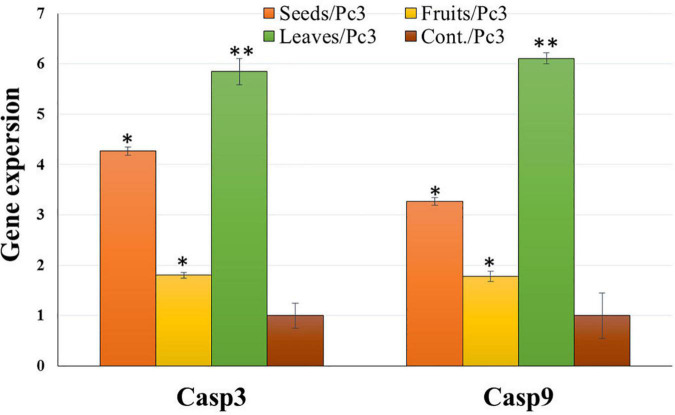
Effect of leaf, seed, and fruit extracts on Casp3 and Casp9 gene expression in the pancreatic cancer cell (Pc3). *P-value was considered statistically significant (*P* < 0.05) and **P-value was considered highly significant (*P* < 0.01).

**FIGURE 14:**
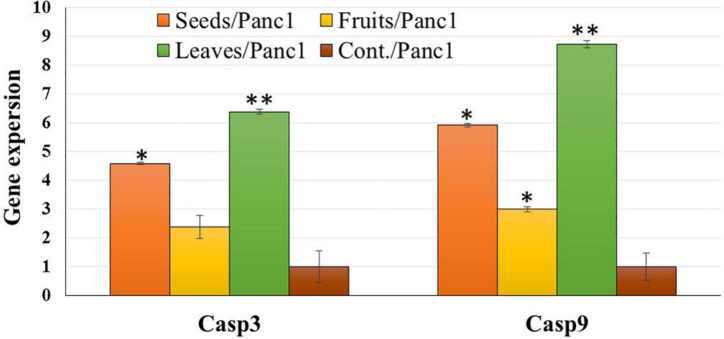
Effect of leaf, seed, and fruit extracts on Casp3 and Casp9 gene expression in the pancreatic cancer cell (Panc1). *P-value was considered statistically significant (*P* < 0.05) and **P-value was considered highly significant (*P* < 0.01).

Treatment of Panc1 and Pc3 IC_50_ of each extract exhibited a significant increase in casp3 and casp9 mRNA levels as compared to untreated controls (Figures 13, 14). Also, the effect of leaf extract was more prominent on the utilized cell lines than on seeds and fruit extracts.

## Discussion

There are multiple natural phytochemicals that could help in protecting the individual from different cancer diseases (52, 53). In addition, functional foods, such as date fruits, have the potential to regulate cancer cell proliferation and differentiation (12). Zhang et al. (54) elaborated those different medicated plants have multiple natural substances such as taxol, vincristine, and vinblastine. Such products have characteristics to compete against cancer cells with the lowest side effects.

In this study, the ethanolic extracts of different KPC parts for Panc1 and Pc3 were evaluated to find the antiproliferative activity in leaves, seeds, and fruits. The results showed that the extracts of that date palm parts gave a defense line to counter cancer cells. It reveals major findings of the tested extracts that might be assorted, like the antiproliferative effect, the apoptotic effect, and the influence on Casp3 and casp9 gene expression. The leaf extract showed the highest antiproliferative activity, higher than seed and fruit extracts. A higher percentage of the phytochemicals was achieved by the KPC leaves extract compared to the other parts. Also, the high presence of phytol in the leaves could explain its high activity against cancer cells. de Alencar et al. (55) mentioned that phytol phytochemical has a very high anticarcinogenic and antitumoral impacts on the different cancer types. This could be explained through the mechanism of action of the phytol prosthetic group presented in KPC leaves. In which, phytol molecule has a high ability to induce ligand-dependent transcription factor called retinoid X receptor (RXR) that functions as a key regulator of cell growth, differentiation, and metabolism of cancer cells (56, 57). Besides, it has been suggested that phytol metabolites have functional impacts on cellular metabolism through activating the uncoupling protein-1 gene transcription (58). This sheds new light on the consequences which might involve excessive activation of phytol targets that have been presented in the leaf extract of KPC.

Furthermore, the results showed that the highest phenolics, flavonoid contents, and antioxidant activity were found in leaf extract, followed by seed and eventually by fruit extracts. It is noteworthy that the leaf extract had improved phenolic content more than the other parts (*P* < 0.01). Significantly, the outcomes found in this study were aligned with Qadoos et al. (59) study. Also, these results showed that different *P. dactylifera* extracts had multiple TPC and TFC stages. Mehraban et al. (28) described that the different concentrations of TPC and TFC are contributing to the used parts of KPC. Also, the presence of large amounts of phenolic compounds in the ethanolic extract showed a high antioxidant agent (60–62). Earlier studies found that palm date has antioxidant potential to suppress free radicals based on its TPC and TFC (63, 64). Also, Zineb et al. (65) reported that dates constitute considered a good source of antioxidants. Al-Farsi et al. (66) investigated the antioxidant activities in Fard, Khasab, and KPC. Their study concluded that KPC had the most activity as compared to other types of dates. Besides, Ajwa and KPC date extracts showed higher polyphenol contents than Sukkari date extract (63).

As a precise colorimetric approach, MTT assay is a quick technique that shows cell growth and cytotoxicity. It also examines the phytochemcials cytotoxicity and could evaluate their impacts on the cancer cells’ proliferation and viability (67). MTT assay stated that extracts could be used as an antiproliferative agent which attacks cancer cells with an appropriate dose. The cytotoxic effect of leaf extract was the highest one, followed by seed and fruit extract. This impact was through the functional groups of phytol that is the part of the chlorophyll dye presented in the leaves extracts. Thakor et al. (56) mentioned that the mechanism of action of phytol cytotoxicity came from its significant impact on the intrinsic pathway enzyme caspase 9 as well as the common executioner caspase 3. Moreover, the cytotoxic effect of Ajwa date pulp against hepatocellular carcinoma (hepG2) was attributed to β-D-glucan as reported by Siddiqui et al. (68). Also, the dates quercetin and kaempferol showed high cytotoxic activity against pc3 cells (69, 70). Another *in vitro* study was conducted by Al-Sayyed et al. (71) and reported that Barhi date palm fruit prevents 7,12-Dimethylbenzathracene (DMBA)-induced mammary cancer. Its outcomes can be correlated with the other two drugs, i.e., tamoxifen and 17-β-estradiol. This effect is caused due to its high phenolic and flavonoid contents.

Also, the high cytotoxic effect of the allocated in the phenolic compounds found in the KPC’s extracts of leaves, seeds, and fruit could be from the pro-oxidant and modification of the redox balance in the Pc3 and Panc1 cancer cells (72, 73). Mirza et al. (70) reported that cancer cells can be controlled through the cell cycle and induction of apoptosis. Additionally, Stewart (74) briefed that the apoptotic morphological changes could explain the cytotoxic effect of date phytochemicals. In which, Panc1 and Pc3 cells that reacted with the selected extracts showed the characteristics of apoptosis. Regarding induction of apoptosis and cell cycle arrest. Flow cytometry results using Annexin V-FITC and PI were presented as a percentage of 786 Annexin V+/PI− or Annexin V+/PI+ to indicate the status of apoptosis and necrosis induced by the 787 test compounds.

In our study, the seed extract caused Pc3 cells to move toward the late apoptosis stage and then induced cancer cell death. Khan et al. (75) reported that Ajwa dates extract tended to induce apoptosis in breast cancer MCF-7 cells by damaging the cancer cells’ DNA. Also, Vessoni et al. (76) reported that if the cell is severely damaged, it can be forwarded to apoptosis to stop the proliferation of cancer cells. This study identified that cell cycle analysis shows a high level of cells found in preG1 and G2/M phases to control cells. The outcomes of this study are validated by the earlier studies which discussed anticancer drugs, i.e., paclitaxel. Such a drug inhibited human tenon’s fibroblast cell proliferation through cell cycle arrest at G2/M phases (77). Also, these findings are in line with a recently published report by Siddiqui (68) on Ajwa dates. Its extracts are helpful to counter HepG2 cells. In addition, their findings showed that extract of KPC date palm inhibited cell proliferation via G2/M phase arrest. Yamashita and Kawanishi (78) and Marvibaigi et al. (79) reported that phenolic and flavonoids contents helped apoptosis and induced the cell cycle arrest through the inhibition of DNA replication.

The effect of KPC date extracts of leaves, seeds, and fruits on the expression levels of apoptosis-related genes (Casp3 and Casp9) was observed among Pnc1 and Pc3 cells. The cysteine protease activation, particularly caspases, is a crucial intracellular regulator of cell apoptosis (80). CASP 3 and CASP 9 genes belong to the cysteine-aspartic acid protease family and play a central role in inducing apoptosis (81). Alenzi et al. (82) stated that caspase 3 has a substantial arbitrator of apoptosis. Hünten et al. (80) reported that it activates caspase 9 through a wide range of activators. Also, apoptosis is activated from two signaling pathways, i.e., extrinsic and intrinsic pathways. The extrinsic pathway is arbitrated through the death receptor. However, the intrinsic pathways are arbitrated to the mitochondrion. Su et al. (83) stated that the caspase gene has a vital role in mitochondrion-mediated apoptosis. Zhang et al. (54) indicated that casp-3 has an activated protease in mammalian cell apoptosis. It came through activation, proteolysis, and hydrolysis of particular substrates, i.e., DNA-dependent protein kinases. Badgujar et al. (84) reported that Casp-3 is triggered by casp-9.

The expression of Casp3 and Casp9 was tested to analyze the mechanism underlying the induction of apoptosis due to treatment with KPC extracts of leaves, seeds, and fruits. Casp3 and Casp9 expression were significantly up-regulated, and the mRNA level was increased among treated cells compared to untreated cells in all treatments with the two utilized cancerous cell lines. This result indicates that the induction of cell death of these lines was in the apoptosis way (85). The up-regulation of Casp3 and Casp9 gene expression suggested that the treatment of Panc1 and Pc3 cells with the tested extracts induces mitochondrial dysfunction by discharging cytochrome c and inducing the intrinsic pathway apoptosis of cancer cells caspases 3 and 9 activations (86). Casp3 and Casp9 gene expression up-regulation might be due to the phytochemical contents in examined plant extracts (87–89). Leaves extract showed the highest effect on the gene expression change, followed by seeds extract and eventually by fruit extract. This result might be due to the different phytochemical contents in different plant parts (90).

## Conclusion

This study concluded that KDC leaves, seeds, and fruit extracts have significant impacts on the Pc3 and Panc1 through their significant impacts on Casp3 and Casp9 gene expressions. The leaves extract showed a high significant (*P* < 0.01) effect in down-regulating Pc3 proliferation as compared to fruits and seeds extracts. Also, Panc1 had the least Casp3 and Casp9 expressions. After treating Panc1 and Pc3 cells with leaves extract, the cell cycle analysis was maximum in G0–G1%. Moreover, the least%PreG1 was found to be 1.39% in the control group of Pc3. Additionally, the percentage of apoptotic and necrotic cells in Panc1 after the treatment with leaf extract was found to be 16.11% in total apoptosis of leaves/Panc1, which was the highest compared to seeds (12.07%), and fruits (5.61%). The IC_50_ of seed extracts on Pc3 had a significantly (*P* < 0.05) lower concentration than Fruit extracts with cytotoxicity of 1.13% compared to the control with 88.63%. Thus, the present results indicated that KPC extracts have antiproliferation activity by inducing apoptosis in the caspase-3 independent pathway and G2/M phases in Panc1 and Pc3 cells. This study is opening the way toward using the phytochemicals of KPC through new technologies like micro and nanoencapsulation for establishing functional bioactive food that could be used for the prevention of prostate and pancreatic cancers. In which, the molecular pathway was confirmed for the related expressed genes. Further *in vivo* studies could help in tracking the changes in Casp3 and Casp9 in the vital body system.

## Data availability statement

The original contributions presented in the study are included in the article/supplementary material, further inquiries can be directed to the corresponding authors.

## Author contributions

HG: conceptualization, methodology, investigation, funding, and writing—original draft preparation. MG: conceptualization, methodology, investigation, formal analysis, writing—original draft preparation, and writing—review and editing. NA: conceptualization, methodology, writing—original draft preparation, and funding. NA-H: formal analysis and investigation. MA: methodology, investigation, and formal analysis. MA-S: investigation and data curation. MAA: methodology, software, and writing—original draft preparation. SA-S: investigation and writing—review and editing. AH: formal analysis, validation, and writing—review and editing. HA: software, data curation, and writing—review and editing. GG: data curation and writing—review and editing. DH: investigation and writing—original draft preparation. All authors have read and agreed to the published version of the manuscript.

## References

[1] Serhani M Essaadi H Kassara K Boutoulout A. Control by viability in a chemotherapy cancer model. *Acta Biotheor.* (2019) 67:177–200. 10.1007/s10441-019-09344-0 30949871

[2] Hussein L Gouda M Buttar HS. Pomegranate, its components and modern deliverable formulations as potential botanicals in the prevention and treatment of various cancers. *Curr Drug Deliv.* (2021) 18:1–15. 10.2174/1567201818666210203180853 33538675

[3] Ajdžanovic V Filipovic B Miljic D Mijatovic S Maksimovic-Ivanic D Miler M Prostate cancer metastasis and soy isoflavones: a dogfight over a bone. *EXCLI J.* (2019) 18:106. 30956643 PMC6449674

[4] Rawla P. Epidemiology of prostate cancer. *World J Oncol.* (2019) 10:63. 10.14740/wjon1191 31068988 PMC6497009

[5] Al-Abdin OZ Al-Beeshi IZ. Prostate cancer in the Arab population: an overview. *Saudi Med J.* (2018) 39:453. 10.15537/smj.2018.5.21986 29738003 PMC6118189

[6] Lv J-M Gouda M El-Din Bekhit A He Y-K Ye X-Q Chen J-C. Identification of novel bioactive proanthocyanidins with potent antioxidant and anti-proliferative activities from kiwifruit leaves. *Food Biosci.* (2022) 46:101554. 10.1016/j.fbio.2022.101554

[7] Wang X Zhang H Chen X. Drug resistance and combating drug resistance in cancer. *Cancer Drug Resist.* (2019) 2:141–60. 10.20517/cdr.2019.10 34322663 PMC8315569

[8] Jiang M Zheng S. Geniposide inhibits non-small cell lung cancer cell migration and angiogenesis by regulating PPARγ/VEGF-A pathway. *Qual Assur Saf Crops Foods.* (2022) 14:46–54. 10.15586/qas.v14i1.1016

[9] Al-Harbi NA Awad NS Alsberi HM Abdein MA. Apoptosis induction, cell cycle arrest and in vitro anticancer potentiality of *Convolvulus spicatus* and *Astragalus vogelii*. *World.* (2019) 8:69–75.

[10] Livingstone TL Beasy G Mills RD Plumb J Needs PW Mithen R Plant bioactives and the prevention of prostate cancer: evidence from human studies. *Nutrients.* (2019) 11:2245. 10.3390/nu11092245 31540470 PMC6769996

[11] Hu Y Xiang X Zhang Y Tian Z Wang L. Aloin promotes oral squamous cell carcinoma cell apoptosis and autophagy through Akt/mTOR pathway. *Qual Assur Saf Crops Foods.* (2022) 14:58–65. 10.15586/qas.v14i2.978

[12] Al Alawi R Alhamdani MSS Hoheisel JD Baqi Y. Antifibrotic and tumor microenvironment modulating effect of date palm fruit (*Phoenix dactylifera* L.) extracts in pancreatic cancer. *Biomed Pharmacother.* (2020) 121:109522. 31675539 10.1016/j.biopha.2019.109522

[13] Gouda M El-Din Bekhit A Tang Y Huang Y Huang L He Y Recent innovations of ultrasound green technology in herbal phytochemistry: a review. *Ultrason Sonochem.* (2021) 73:105538. 10.1016/j.ultsonch.2021.105538 33819867 PMC8048006

[14] Hosseini A Ghorbani A. Cancer therapy with phytochemicals: evidence from clinical studies. *Avicenna J Phytomed.* (2015) 5:84. 25949949 PMC4418057

[15] Hikal DM Awad NS Abdein MA. The anticancer activity of cashew (*Anacardium occidentale*) and almond (*Prunus dulcis*) kernels. *Adv Environ Biol.* (2017) 11:31–41.

[16] Qin G Yi S. Genkwanin improves inflammatory injury in rats with septic lung injury by regulating NF-κB signaling pathway. *Qual Assur Saf Crops Foods.* (2022) 14:66–73. 10.15586/qas.v14i2.991

[17] Rodríguez M Estrela J Ortega Á. Natural polyphenols and apoptosis induction in cancer therapy. *J Carcinog Mutag.* (2013) S6:004. 10.4172/2157-2518.S6-004

[18] El-Kafrawy TM Ghazzawy H Ahmed N Hikal DM. Evaluation of quality and storability of “Sewy” date palm cv. in different production areas in Egypt. *Am Eurasian J Sustain Agric.* (2018) 12:30–9.

[19] Ghazzawy HS Mahdy EM Ali-Dinar HM El-Beltagi HS. Impact of geographical distribution on genetic variation of two date palm cultivars in arid regions. *Fresenius Environ Bull.* (2021) 30:11513–23.

[20] Ghazzawy H Alhajhoj M Munir M. In-vitro somatic embryogenesis response of date palm cv. Sukkary to sucrose and activated charcoal concentrations. *J Appl Hort.* (2017) 19:91–5. 10.37855/jah.2017.v19i02.16

[21] Brima EI. Evaluation of selected essential elements in khalas dates from date palm determined by inductively coupled plasma-mass spectrometry. *Int J Anal Chem.* (2019) 2019:7619692. 10.1155/2019/7619692 31275391 PMC6582846

[22] Fard NN Noorbazargan H Mirzaie A Hedayati Ch M Moghimiyan Z Rahimi A. Biogenic synthesis of AgNPs using *Artemisia Oliveriana* extract and their biological activities for an effective treatment of lung cancer. *Artif Cells Nanomed Biotechnol.* (2018) 46(Suppl. 3):S1047–58. 10.1080/21691401.2018.1528983 30479160

[23] Ahmed MB Hasona NA-S Selemain HA-H. Protective effects of extract from dates (*Phoenix dactylifera* L.) and ascorbic acid on thioacetamide-induced hepatotoxicity in rats. *Iran J Pharm Res.* (2008) 7:193–201.

[24] Pujari RR Vyawahare NS Kagathara VG. Evaluation of antioxidant and neuroprotective effect of date palm (*Phoenix dactylifera* L.) against bilateral common carotid artery occlusion in rats. *Indian J Exp Biol.* (2011) 49:627–33. 21870431

[25] Souli A Sebai H Rtibi K Chehimi L Sakly M Amri M Effects of dates pulp extract and palm sap (*Phoenix dactylifera* L.) on gastrointestinal transit activity in healthy rats. *J Med Food.* (2014) 17:782–6. 10.1089/jmf.2013.0112 24611963 PMC4098977

[26] Mard SA Jalalvand K Jafarinejad M Balochi H Naseri MKG. Evaluation of the antidiabetic and antilipaemic activities of the hydroalcoholic extract of *Phoenix dactylifera* palm leaves and its fractions in alloxan-induced diabetic rats. *Malays J Med Sci.* (2010) 17:4. PMC3216186 22135555

[27] Abuelgassim AO. Serum concentrations of glucose and lipids in alloxan diabetic rats. *Pak J Biol Sci.* (2010) 13:1141–5. 10.3923/pjbs.2010.1141.1145 21313891

[28] Mehraban F Jafari M Toori MA Sadeghi H Joodi B Mostafazade M Effects of date palm pollen (*Phoenix dactylifera* L.) and *Astragalus ovinus* on sperm parameters and sex hormones in adult male rats. *Iran J Reprod Med.* (2014) 12:705–10. 25469129 PMC4248157

[29] Abu-Elteen KH. Effects of date extract on adhesion of *Candida* species to human buccal epithelial cells in vitro. *J Oral Pathol Med.* (2000) 29:200–5. 10.1034/j.1600-0714.2000.290502.x 10801036

[30] Smaoui S Ben Hlima H Fourati M Elhadef K Ennouri K Mellouli L. Multiobjective optimization of *Phoenix dactylifera* L. seeds extraction: mixture design methodology for phytochemical contents and antibacterial activity. *J Food Proc Preserv.* (2020) 44:e14822. 10.1111/jfpp.14822

[31] Eid N Enani S Walton G Corona G Costabile A Gibson G The impact of date palm fruits and their component polyphenols, on gut microbial ecology, bacterial metabolites and colon cancer cell proliferation. *J Nutr Sci.* (2014) 3:e46. 10.1017/jns.2014.16 26101614 PMC4473134

[32] Chakroun M Khemakhem B Mabrouk HB El Abed H Makni M Bouaziz M Evaluation of anti-diabetic and anti-tumoral activities of bioactive compounds from *Phoenix dactylifera* L’s leaf: in vitro and in vivo approach. *Biomed Pharmacother.* (2016) 84:415–22. 10.1016/j.biopha.2016.09.062 27668542

[33] Abou-Elella F Mourad R. Anticancer and anti-oxidant potentials of ethanolic extracts of *Phoenix dactylifera, Musa acuminata* and *Cucurbita maxima*. *Res J Pharm Biol Chem Sci.* (2015) 6:710.

[34] Khan F Khan TJ Kalamegam G Pushparaj PN Chaudhary A Abuzenadah A Anti-cancer effects of Ajwa dates (*Phoenix dactylifera* L.) in diethylnitrosamine induced hepatocellular carcinoma in Wistar rats. *BMC Complement Altern Med.* (2017) 17:418. 10.1186/s12906-017-1926-6 28830415 PMC5567468

[35] Rahmani AH Aly SM Ali H Babiker AY Srikar S. Therapeutic effects of date fruits (*Phoenix dactylifera*) in the prevention of diseases via modulation of anti-inflammatory, anti-oxidant and anti-tumour activity. *Int J Clin Exp Med.* (2014) 7:483. 24753740 PMC3992385

[36] Parvin S Easmin D Sheikh A Biswas M Sharma SCD Jahan MGS Nutritional analysis of date fruits (*Phoenix dactylifera* L.) in perspective of Bangladesh. *Am J Life Sci.* (2015) 3:274–8. 10.11648/j.ajls.20150304.14

[37] Gasim AA. Changes in sugar quality and mineral elements during fruit development in five date palm cultivars in AI-Madinah AI-Munawwarah. *Science.* (1994) 6:29–36. 10.4197/Sci.6-1.3

[38] Al-Alawi RA Al-Mashiqri JH Al-Nadabi JS Al-Shihi BI Baqi Y. Date palm tree (*Phoenix dactylifera* L.): natural products and therapeutic options. *Front Plant Sci.* (2017) 8:845. 10.3389/fpls.2017.00845 28588600 PMC5440559

[39] AOAC. *Official Methods of Analysis.* 17th ed. Gaithersburg, MD: The Association of Official Analytical Chemists (2000). p. 65–692.

[40] Pieme CA Kumar SG Dongmo MS Moukette BM Boyoum FF Ngogang JY Antiproliferative activity and induction of apoptosis by *Annona muricata* (Annonaceae) extract on human cancer cells. *BMC Complement Altern Med.* (2014) 14:516. 10.1186/1472-6882-14-516 25539720 PMC4324658

[41] Charoensin S. Antioxidant and anticancer activities of *Moringa oleifera* leaves. *J Med Plants Res.* (2014) 8:318–25. 10.5897/JMPR2013.5353

[42] Bray F Ferlay J Soerjomataram I Siegel RL Torre LA Jemal A. Global cancer statistics 2018: GLOBOCAN estimates of incidence and mortality worldwide for 36 cancers in 185 countries. *CA Cancer J Clin.* (2018) 68:394–424. 10.3322/caac.21492 30207593

[43] El Sherif F Albotnoor N Yap Y-K Meligy A Khattab S. Enhanced bioactive compounds composition in *Lavandula* officinalis in-vitro plantlets using NaCl and *Moringa oleifera*, Aloe vera and *Spirulina platensis* extracts. *Ind Crops Prod.* (2020) 157:112890. 10.1016/j.indcrop.2020.112890

[44] Pumiputavon K Chaowasku T Saenjum C Osathanunkul M Wungsintaweekul B Chawansuntati K Cell cycle arrest and apoptosis induction by methanolic leaves extracts of four Annonaceae plants. *BMC Complement Altern Med.* (2017) 17:294. 10.1186/s12906-017-1811-3 28583139 PMC5460496

[45] Azadmehr A Hajiaghaee R Mazandarani M. Induction of apoptosis and G2/M cell cycle arrest by *Scrophularia* striata in a human leukaemia cell line. *Cell Prolif.* (2013) 46:637–43. 10.1111/cpr.12074 24460717 PMC6496267

[46] Guido F Behija SE Manel I Nesrine Z Ali F Mohamed H Chemical and aroma volatile compositions of date palm (*Phoenix dactylifera* L.) fruits at three maturation stages. *Food Chem.* (2011) 127:1744–54. 10.1016/j.foodchem.2011.02.051

[47] Al-Shahib W Marshall RJ. The fruit of the date palm: its possible use as the best food for the future? *Int J Food Sci Nutr.* (2003) 54:247–59. 10.1080/09637480120091982 12850886

[48] Al-Farsi M Alasalvar C Al-Abid M Al-Shoaily K Al-Amry M Al-Rawahy F. Compositional and functional characteristics of dates, syrups, and their by-products. *Food Chem.* (2007) 104:943–7. 10.1016/j.foodchem.2006.12.051

[49] Sayed DR Aly MHA Sayed GH. Improving quality of date palm (*Phoenix dactylifera* L.) fruits CVS. Khalas and Sagae under different climate by spraying of date palm pollen grains extract. *Int J Biosci.* (2018) 12:56–69. 10.12692/ijb/12.3.56-69

[50] Ghazzawy H Alhajhoj M Sallam A Munir M. Impact of chemical thinning to improve fruit characteristics of date palm cultivar Khalas. *Iraqi J Agric Sci.* (2019) 50:1361–8. 10.36103/ijas.v50i5.802

[51] Munir M Alhajhoj MR Sallam AA Ghazzawy HS Al-Bahigan AM. Fruit yield and quality response of date palm cultivar Khalas to female inflorescence receptivity varied by pollination days. *Plant Arch.* (2020) 20:4007–14.

[52] Cao Z Ma J Shen X. Alpinetin suppresses cell proliferation and metastasis in osteosarcoma by inhibiting PI3K/AKT and ERK pathways. *Qual Assur Saf Crops Foods.* (2022) 14:112–8. 10.15586/qas.v14i2.1084

[53] Wang G Zhang Y Xiaolu Z Mei Y Ma X Liu X. Norcantharidin alleviates cyclophosphamide-induced immunosuppression via circBCL2L1/miR-30c-3-3p/TRAF6 axis. *Qual Assur Saf Crops Foods.* (2022) 14:94–102. 10.15586/qas.v14i3.1103

[54] Zhang Y-X Kong C-Z Wang H-Q Wang L-H Xu C-L Sun Y-H. Phosphorylation of Bcl-2 and activation of caspase-3 via the c-Jun N-terminal kinase pathway in ursolic acid-induced DU145 cells apoptosis. *Biochimie.* (2009) 91:1173–9. 10.1016/j.biochi.2009.06.010 19545597

[55] de Alencar M Islam MT de Lima RMT Paz M Dos Reis AC da Mata A Phytol as an anticarcinogenic and antitumoral agent: an in vivo study in swiss mice with DMBA-Induced breast cancer. *IUBMB Life.* (2019) 71:200–12. 10.1002/iub.1952 30394663

[56] Thakor P Subramanian RB Thakkar SS Ray A Thakkar VR. Phytol induces ROS mediated apoptosis by induction of caspase 9 and 3 through activation of TRAIL, FAS and TNF receptors and inhibits tumor progression factor Glucose 6 phosphate dehydrogenase in lung carcinoma cell line (A549). *Biomed Pharmacother.* (2017) 92:491–500. 10.1016/j.biopha.2017.05.066 28575806

[57] Lefebvre P Benomar Y Staels B. Retinoid X receptors: common heterodimerization partners with distinct functions. *Trends Endocrinol Metab.* (2010) 21:676–83. 10.1016/j.tem.2010.06.009 20674387

[58] Kitareewan S Burka LT Tomer KB Parker CE Deterding LJ Stevens RD Phytol metabolites are circulating dietary factors that activate the nuclear receptor RXR. *Mol Biol Cell.* (1996) 7:1153–66. 10.1091/mbc.7.8.1153 8856661 PMC275969

[59] Qadoos HA Dhafari HS Al Marzooqi DA Kumarappan A Nazir A. Phenolic content and antimicrobial activities of date palm (*Phoenix dactylifera* L.) fruits and leaves. *Food Biol.* (2017) 6:11–5. 10.19071/fbiol.2017.v6.3154

[60] Saha S Barua B Sikdar D. Phytochemical screening, phenolic content and antioxidant activity of wild date palm (*Phoenix sylvestris* Roxb.) fruit extracted with different solvents. *Int Food Res J.* (2017) 24:2534–42.

[61] John JA Shahidi F. Phenolic content, antioxidant and anti-inflammatory activities of seeds and leaves of date palm (*Phoenix dactylifera* L.). *J Food Bioact.* (2019) 5:120–30. 10.31665/JFB.2019.5179

[62] Radfar R Farhoodi M Ghasemi I Khaneghah AM Shahraz F Hosseini H. Assessment of phenolic contents and antioxidant and antibacterial activities of extracts from four varieties of Iranian date Palm (*Phoenix dactylifera* L.) seeds. *Appl Food Biotechnol.* (2019) 6:173–84.

[63] Saleh EA Tawfik MS Abu-Tarboush HM. Phenolic contents and antioxidant activity of various date palm (*Phoenix dactylifera* L.) fruits from Saudi Arabia. *Food Nutr Sci.* (2011) 2:1134–41. 10.4236/fns.2011.210152

[64] Ragab AR Elkablawy MA Sheik BY Baraka HN. Antioxidant and tissue-protective studies on Ajwa extract: dates from al-Madinah al-Monwarah, Saudia Arabia. *J Environ Anal Toxicol.* (2013) 3:2161–525. 10.4172/2161-0525.1000163

[65] Zineb G Boukouada M Djeridane A Saidi M Yousfi M. Screening of antioxidant activity and phenolic compounds of various date palm (*Phoenix dactylifera*) fruits from Algeria. *Mediterr J Nutr Metab.* (2012) 5:119–26. 10.1007/s12349-011-0082-7

[66] Al-Farsi M Alasalvar C Morris A Baron M Shahidi F. Comparison of antioxidant activity, anthocyanins, carotenoids, and phenolics of three native fresh and sun-dried date (*Phoenix dactylifera* L.) varieties grown in Oman. *J Agric Food Chem.* (2005) 53:7592–9. 10.1021/jf050579q 16159191

[67] Jaszczyszyn A Ga̧siorowski K. Limitations of the MTT assay in cell viability testing. *Adv Clin Exp Med.* (2008) 17:525–9.

[68] Siddiqui S Ahmad R Khan MA Upadhyay S Husain I Srivastava AN. Cytostatic and anti-tumor potential of Ajwa date pulp against human hepatocellular carcinoma HepG2 cells. *Sci Rep.* (2019) 9:245. 10.1038/s41598-018-36475-0 30664656 PMC6341075

[69] Abdul-Hamid NA Mediani A Maulidiani M Shadid K Ismail IS Abas F Metabolite characterization of different palm date varieties and the correlation with their NO inhibitory activity, texture and sweetness. *J Food Sci Technol.* (2018) 55:1541–51. 10.1007/s13197-018-3073-6 29606769 PMC5876226

[70] Mirza MB Elkady AI Al-Attar AM Syed FQ Mohammed FA Hakeem KR. Induction of apoptosis and cell cycle arrest by ethyl acetate fraction of *Phoenix dactylifera* L.(Ajwa dates) in prostate cancer cells. *J Ethnopharmacol.* (2018) 218:35–44. 10.1016/j.jep.2018.02.030 29476962

[71] Al-Sayyed HF Takruri HR Shomaf MS. The effect of date palm fruit (*Phoenix dactylifera* L.) on 7, 12-dimethylbenz (α) anthracene (DMBA)-induced mammary cancer in rats. *Res Opin Anim Vet Sci.* (2014) 4:11–8. 10.3233/MNM-140001

[72] Massaoka MH Matsuo AL Figueiredo CR Farias CF Girola N Arruda DC Jacaranone induces apoptosis in melanoma cells via ROS-mediated downregulation of Akt and p38 MAPK activation and displays antitumor activity in vivo. *PLoS One.* (2012) 7:e38698. 10.1371/journal.pone.0038698 22701695 PMC3368838

[73] Aggarwal V Tuli HS Varol A Thakral F Yerer MB Sak K Role of reactive oxygen species in cancer progression: molecular mechanisms and recent advancements. *Biomolecules.* (2019) 9:735. 10.3390/biom9110735 31766246 PMC6920770

[74] Stewart BW. Mechanisms of apoptosis: integration of genetic, biochemical, and cellular indicators. *J Natl Cancer Inst.* (1994) 86:1286–96. 10.1093/jnci/86.17.1286 8064887

[75] Khan F Ahmed F Pushparaj PN Abuzenadah A Kumosani T Barbour E Ajwa date (*Phoenix dactylifera* L.) extract inhibits human breast adenocarcinoma (MCF7) cells in vitro by inducing apoptosis and cell cycle arrest. *PLoS One.* (2016) 11:e0158963. 10.1371/journal.pone.0158963 27441372 PMC4956039

[76] Vessoni A Filippi-Chiela E Menck CF Lenz G. Autophagy and genomic integrity. *Cell Death Different.* (2013) 20:1444–54. 10.1038/cdd.2013.103 23933813 PMC3792426

[77] Chen N Guo D Guo Y Sun Y Bi H Ma X. Paclitaxel inhibits cell proliferation and collagen lattice contraction via TGF-β signaling pathway in human tenon’s fibroblasts in vitro. *Eur J Pharmacol.* (2016) 777:33–40. 10.1016/j.ejphar.2016.02.059 26930229

[78] Yamashita N Kawanishi S. Distinct mechanisms of DNA damage in apoptosis induced by quercetin and luteolin. *Free Radic Res.* (2000) 33:623–33. 10.1080/10715760000301141 11200093

[79] Marvibaigi M Amini N Supriyanto E Abdul Majid FA Kumar Jaganathan S Jamil S Antioxidant activity and ROS-dependent apoptotic effect of *Scurrula ferruginea* (Jack) danser methanol extract in human breast cancer cell MDA-MB-231. *PLoS One.* (2016) 11:e0158942. 10.1371/journal.pone.0158942 27410459 PMC4943642

[80] Hünten S Siemens H Kaller M Hermeking H. The p53/microRNA network in cancer: experimental and bioinformatics approaches. *Adv Exp Med Biol.* (2013) 774:77–101. 10.1007/978-94-007-5590-1_5 23377969

[81] Safarzadeh E Delazar A Kazemi T Orangi M Shanehbandi D Esnaashari S The cytotoxic and apoptotic effects of *Scrophularia* atropatana extracts on human breast cancer cells. *Adv Pharm Bull.* (2017) 7:381. 10.15171/apb.2017.046 29071220 PMC5651059

[82] Alenzi FQ Alenazi BQ Al-Anazy FH Mubaraki AM Salem ML Al-Jabri AA The role of caspase activation and mitochondrial depolarisation in cultured human apoptotic eosinophils. *Saudi J Biol Sci.* (2010) 17:29–36. 10.1016/j.sjbs.2009.12.005 23961055 PMC3730707

[83] Su Z Yang Z Xu Y Chen Y Yu Q. Apoptosis, autophagy, necroptosis, and cancer metastasis. *Mol Cancer.* (2015) 14:48. 10.1186/s12943-015-0321-5 25743109 PMC4343053

[84] Badgujar NV Mistry KN Rank DN Joshi CG. Screening of antiproliferative activity mediated through apoptosis pathway in human non-small lung cancer A-549 cells by active compounds present in medicinal plants. *Asian Pac J Trop Med.* (2018) 11:666. 10.4103/1995-7645.248338

[85] Och A Zalewski D Komsta Ł Kołodziej P Kocki J Bogucka-Kocka A. Cytotoxic and proapoptotic activity of sanguinarine, berberine, and extracts of *Chelidonium majus* L. and *Berberis thunbergii* DC. Toward hematopoietic cancer cell lines. *Toxins.* (2019) 11:485. 10.3390/toxins11090485 31443589 PMC6784183

[86] Emam MA Khattab HI Hegazy MG. Assessment of anticancer activity of *Pulicaria undulata* on hepatocellular carcinoma HepG2 cell line. *Tumor Biol.* (2019) 41:1010428319880080. 10.1177/1010428319880080 31603389

[87] Li S Dong P Wang J Zhang J Gu J Wu X Icariin, a natural flavonol glycoside, induces apoptosis in human hepatoma SMMC-7721 cells via a ROS/JNK-dependent mitochondrial pathway. *Cancer Lett.* (2010) 298:222–30. 10.1016/j.canlet.2010.07.009 20674153

[88] Kumar M Kaur V Kumar S Kaur S. Phytoconstituents as apoptosis inducing agents: strategy to combat cancer. *Cytotechnology.* (2016) 68:531–63. 10.1007/s10616-015-9897-2 26239338 PMC4960184

[89] Rezaei PF Fouladdel S Hassani S Yousefbeyk F Ghaffari SM Amin G Induction of apoptosis and cell cycle arrest by pericarp polyphenol-rich extract of Baneh in human colon carcinoma HT29 cells. *Food Chem Toxicol.* (2012) 50:1054–9. 10.1016/j.fct.2011.11.012 22119783

[90] Saxena H Deshmukh A Kakkar A Singh N. Phytochemical screening and assessment of secondary metabolites in different plant parts of *Solanum xanthocarpum*: a Dashmool species. *Indian J Trop Biodiv.* (2014) 22:164–9.

